# (Benzohydrazidato-κ^2^ *N*′,*O*)­[2-(benzoyl­hydrazono-κ^2^ *N*,*O*)propion­ato-κ*O*]oxido­vanadium(V)

**DOI:** 10.1107/S1600536809009520

**Published:** 2009-03-25

**Authors:** Hon Wee Wong, Kong Mun Lo, Seik Weng Ng

**Affiliations:** a Department of Chemistry, University of Malaya, 50603 Kuala Lumpur, Malaysia

## Abstract

The V^V^ atom in the title compound, [VO(C_7_H_7_N_2_O)(C_10_H_8_N_2_O_3_)], is *N*,*O*-chelated by the benzohydrazidate anion and *O*,*N*,*O*′-chelated by the 2-(benzoyl­hydrazono)propionate dianion. The distorted octa­hedral trans-N_2_O_4_ coordination geometry is completed by the vandadyl O atom. Mol­ecules are linked by N—H⋯O hydrogen bonds into a supra­molecular chain structure parallel to [010].

## Related literature

For other benzoyl­hydrazido–oxovanadium compounds, see: Kopka & Mattes (1995 ▶); Sundheim *et al.* (1994 ▶).
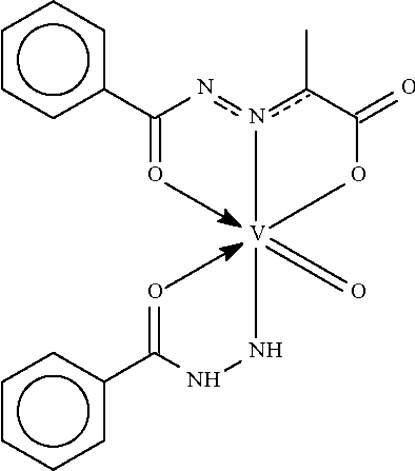


## Experimental

### 

#### Crystal data

[V(C_7_H_7_N_2_O)(C_10_H_8_N_2_O_3_)O]
*M* *_r_* = 406.27
Monoclinic, 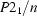

*a* = 10.9424 (2) Å
*b* = 6.2384 (1) Å
*c* = 25.7215 (5) Å
β = 96.603 (1)°
*V* = 1744.18 (5) Å^3^
*Z* = 4
Mo *K*α radiation
μ = 0.61 mm^−1^
*T* = 123 K
0.35 × 0.10 × 0.03 mm

#### Data collection

Bruker SMART APEX diffractometer
Absorption correction: multi-scan (*SADABS*; Sheldrick, 1996 ▶) *T*_min_ = 0.816, *T*_max_ = 0.982
11614 measured reflections
4010 independent reflections
3330 reflections with *I* > 2σ(*I*)
*R*_int_ = 0.027

#### Refinement

*R*[*F* ^2^ > 2σ(*F* ^2^)] = 0.034
*wR*(*F* ^2^) = 0.091
*S* = 1.00
4010 reflections
253 parameters
2 restraints
H atoms treated by a mixture of independent and constrained refinement
Δρ_max_ = 0.39 e Å^−3^
Δρ_min_ = −0.38 e Å^−3^

### 

Data collection: *APEX2* (Bruker, 2008 ▶); cell refinement: *SAINT* (Bruker, 2008 ▶); data reduction: *SAINT*; program(s) used to solve structure: *SHELXS97* (Sheldrick, 2008 ▶); program(s) used to refine structure: *SHELXL97* (Sheldrick, 2008 ▶); molecular graphics: *X-SEED* (Barbour, 2001 ▶); software used to prepare material for publication: *publCIF* (Westrip, 2009 ▶).

## Supplementary Material

Crystal structure: contains datablocks global, I. DOI: 10.1107/S1600536809009520/tk2396sup1.cif

Structure factors: contains datablocks I. DOI: 10.1107/S1600536809009520/tk2396Isup2.hkl

Additional supplementary materials:  crystallographic information; 3D view; checkCIF report

## Figures and Tables

**Table 1:** Hydrogen-bond geometry (Å, °)

*D*—H⋯*A*	*D*—H	H⋯*A*	*D*⋯*A*	*D*—H⋯*A*
N3—H3⋯O4^i^	0.87 (1)	1.97 (1)	2.823 (2)	164 (2)
N4—H4⋯O3^i^	0.88 (1)	2.05 (1)	2.861 (2)	154 (2)

Symmetry code: (i) 
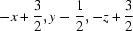
.

**Fig. 1.**
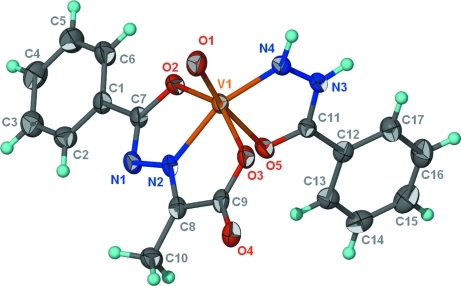
Thermal ellipsoid plot (Barbour, 2001) of VO(C7H7N2O)(C10H8N2O3) at the 70% probability level. Hydrogen atoms are drawn as spheres of arbitrary radius.

## supplementary crystallographic information

### Experimental

2-[Benzoylhydrazono]propionic acid (0.66 g, 3.2 mmol), prepared from the condensation reaction of benzhydrazide and pyruvic acid, was dissolved in of ethanol (50 ml). It was then mixed with vanadyl sulfate (0.26 g, 1.6 mmol) in distilled water (20 ml) and the mixture was heated for 5 h. Upon slow evaporation of the filtrate, red crystals formed.

### Refinement

Carbon-bound H-atoms were placed in calculated positions (C—H 0.95 to 0.98 Å) and were included in the refinement in the riding model approximation, with *U*(H) set to 1.2-1.5*U*(C).

The nitrogen-bound H-atoms were located in a difference Fourier map, and were refined with a distance restraint of N–H 0.88±0.01 Å; their U_iso_ values were freely refined.

### Crystal data

**Table:**

[V(C_7_H_7_N_2_O)(C_10_H_8_N_2_O_3_)O]	*F*(000) = 832
*M**_r_* = 406.27	*D*_x_ = 1.547 Mg m^−^^3^
Monoclinic, *P*2_1_/*n*	Mo *K*α radiation, λ = 0.71073 Å
Hall symbol: -P 2yn	Cell parameters from 3550 reflections
*a* = 10.9424 (2) Å	θ = 2.9–28.0°
*b* = 6.2384 (1) Å	µ = 0.61 mm^−^^1^
*c* = 25.7215 (5) Å	*T* = 123 K
β = 96.603 (1)°	Prism, red
*V* = 1744.18 (5) Å^3^	0.35 × 0.10 × 0.03 mm
*Z* = 4	

### Data collection

**Table:**

Bruker SMART APEX diffractometer	4010 independent reflections
Radiation source: fine-focus sealed tube	3330 reflections with *I* > 2σ(*I*)
graphite	*R*_int_ = 0.027
ω scans	θ_max_ = 27.5°, θ_min_ = 1.6°
Absorption correction: multi-scan (*SADABS*; Sheldrick, 1996)	*h* = −14→14
*T*_min_ = 0.816, *T*_max_ = 0.982	*k* = −8→7
11614 measured reflections	*l* = −32→33

### Refinement

**Table:**

Refinement on *F*^2^	Primary atom site location: structure-invariant direct methods
Least-squares matrix: full	Secondary atom site location: difference Fourier map
*R*[*F*^2^ > 2σ(*F*^2^)] = 0.034	Hydrogen site location: inferred from neighbouring sites
*wR*(*F*^2^) = 0.091	H atoms treated by a mixture of independent and constrained refinement
*S* = 1.00	*w* = 1/[σ^2^(*F*_o_^2^) + (0.0443*P*)^2^ + 0.9013*P*] where *P* = (*F*_o_^2^ + 2*F*_c_^2^)/3
4010 reflections	(Δ/σ)_max_ = 0.001
253 parameters	Δρ_max_ = 0.39 e Å^−^^3^
2 restraints	Δρ_min_ = −0.38 e Å^−^^3^

### Fractional atomic coordinates and isotropic or equivalent isotropic displacement parameters (Å^2^)

**Table:**

	*x*	*y*	*z*	*U*_iso_*/*U*_eq_	
V1	0.63939 (3)	0.51362 (5)	0.667678 (11)	0.02025 (10)	
O1	0.71456 (13)	0.3013 (2)	0.65899 (5)	0.0310 (3)	
O2	0.47660 (11)	0.4125 (2)	0.63552 (5)	0.0252 (3)	
O3	0.77761 (11)	0.7223 (2)	0.66978 (4)	0.0227 (3)	
O4	0.87206 (11)	0.9694 (2)	0.62563 (5)	0.0250 (3)	
O5	0.53800 (11)	0.7850 (2)	0.69541 (4)	0.0229 (3)	
N1	0.49712 (13)	0.6388 (3)	0.56603 (5)	0.0230 (3)	
N2	0.60460 (13)	0.6815 (2)	0.59808 (5)	0.0200 (3)	
N3	0.57784 (13)	0.6090 (2)	0.76970 (5)	0.0204 (3)	
H3	0.582 (2)	0.584 (4)	0.8033 (4)	0.039 (6)*	
N4	0.63345 (14)	0.4678 (2)	0.73974 (6)	0.0213 (3)	
H4	0.6650 (17)	0.363 (3)	0.7597 (7)	0.029 (6)*	
C1	0.32064 (16)	0.4069 (3)	0.56296 (7)	0.0235 (4)	
C2	0.26918 (17)	0.4956 (3)	0.51595 (7)	0.0271 (4)	
H2	0.3101	0.6096	0.5006	0.033*	
C3	0.15882 (18)	0.4191 (3)	0.49134 (8)	0.0316 (4)	
H3A	0.1242	0.4805	0.4592	0.038*	
C4	0.09839 (18)	0.2527 (3)	0.51358 (8)	0.0346 (5)	
H4A	0.0221	0.2011	0.4968	0.041*	
C5	0.14961 (19)	0.1621 (4)	0.56019 (8)	0.0366 (5)	
H5	0.1086	0.0477	0.5754	0.044*	
C6	0.26067 (19)	0.2380 (3)	0.58478 (8)	0.0330 (5)	
H6	0.2959	0.1747	0.6166	0.040*	
C7	0.43810 (16)	0.4902 (3)	0.58978 (7)	0.0221 (4)	
C8	0.67646 (16)	0.8356 (3)	0.58852 (7)	0.0217 (4)	
C9	0.78589 (16)	0.8481 (3)	0.62982 (6)	0.0212 (4)	
C10	0.65613 (17)	0.9934 (3)	0.54563 (7)	0.0273 (4)	
H10A	0.6174	0.9223	0.5139	0.041*	
H10B	0.7352	1.0546	0.5388	0.041*	
H10C	0.6022	1.1080	0.5557	0.041*	
C11	0.52823 (15)	0.7794 (3)	0.74341 (6)	0.0191 (3)	
C12	0.46480 (15)	0.9478 (3)	0.77016 (7)	0.0207 (4)	
C13	0.41422 (15)	1.1174 (3)	0.73968 (7)	0.0235 (4)	
H13	0.4241	1.1237	0.7035	0.028*	
C14	0.34966 (17)	1.2765 (3)	0.76211 (8)	0.0297 (4)	
H14	0.3142	1.3913	0.7413	0.036*	
C15	0.33666 (18)	1.2683 (3)	0.81518 (8)	0.0341 (5)	
H15	0.2914	1.3766	0.8305	0.041*	
C16	0.38955 (18)	1.1029 (4)	0.84576 (8)	0.0335 (5)	
H16	0.3821	1.1002	0.8822	0.040*	
C17	0.45334 (17)	0.9412 (3)	0.82367 (7)	0.0275 (4)	
H17	0.4889	0.8270	0.8447	0.033*	

### Atomic displacement parameters (Å^2^)

**Table:**

	*U*^11^	*U*^22^	*U*^33^	*U*^12^	*U*^13^	*U*^23^
V1	0.02461 (16)	0.02017 (17)	0.01501 (15)	0.00332 (12)	−0.00179 (11)	−0.00063 (11)
O1	0.0386 (8)	0.0277 (7)	0.0250 (7)	0.0083 (6)	−0.0030 (6)	−0.0049 (6)
O2	0.0300 (7)	0.0264 (7)	0.0178 (6)	−0.0041 (6)	−0.0026 (5)	0.0027 (5)
O3	0.0242 (6)	0.0258 (7)	0.0172 (6)	0.0014 (5)	−0.0019 (5)	−0.0025 (5)
O4	0.0224 (6)	0.0308 (7)	0.0215 (6)	−0.0022 (5)	0.0014 (5)	−0.0059 (5)
O5	0.0279 (6)	0.0242 (7)	0.0165 (6)	0.0053 (5)	0.0026 (5)	0.0028 (5)
N1	0.0215 (7)	0.0289 (8)	0.0172 (7)	−0.0031 (6)	−0.0030 (6)	0.0004 (6)
N2	0.0206 (7)	0.0252 (8)	0.0137 (6)	0.0013 (6)	−0.0007 (5)	−0.0025 (6)
N3	0.0226 (7)	0.0220 (8)	0.0160 (7)	0.0002 (6)	0.0002 (6)	0.0015 (6)
N4	0.0241 (7)	0.0199 (8)	0.0190 (7)	0.0034 (6)	−0.0016 (6)	0.0014 (6)
C1	0.0243 (9)	0.0263 (9)	0.0196 (8)	−0.0021 (7)	0.0010 (7)	−0.0026 (7)
C2	0.0265 (9)	0.0267 (10)	0.0273 (9)	−0.0012 (8)	−0.0003 (7)	0.0026 (8)
C3	0.0283 (10)	0.0350 (11)	0.0292 (10)	0.0044 (9)	−0.0060 (8)	−0.0004 (9)
C4	0.0261 (10)	0.0374 (12)	0.0386 (11)	−0.0060 (9)	−0.0035 (8)	−0.0076 (9)
C5	0.0387 (11)	0.0377 (12)	0.0330 (11)	−0.0150 (10)	0.0019 (9)	−0.0001 (9)
C6	0.0381 (11)	0.0372 (12)	0.0224 (9)	−0.0108 (9)	−0.0021 (8)	0.0017 (8)
C7	0.0256 (9)	0.0230 (9)	0.0173 (8)	0.0005 (7)	0.0007 (7)	−0.0019 (7)
C8	0.0229 (8)	0.0244 (9)	0.0176 (8)	0.0020 (7)	0.0016 (7)	−0.0030 (7)
C9	0.0222 (8)	0.0236 (9)	0.0176 (8)	0.0049 (7)	0.0009 (6)	−0.0065 (7)
C10	0.0277 (9)	0.0282 (10)	0.0249 (9)	−0.0042 (8)	−0.0024 (7)	0.0036 (8)
C11	0.0180 (8)	0.0206 (8)	0.0180 (8)	−0.0017 (7)	−0.0004 (6)	0.0006 (7)
C12	0.0154 (8)	0.0232 (9)	0.0236 (9)	−0.0025 (7)	0.0036 (6)	−0.0017 (7)
C13	0.0186 (8)	0.0254 (9)	0.0266 (9)	−0.0025 (7)	0.0027 (7)	0.0008 (7)
C14	0.0227 (9)	0.0252 (10)	0.0408 (11)	0.0006 (8)	0.0023 (8)	0.0005 (8)
C15	0.0262 (10)	0.0337 (11)	0.0436 (12)	0.0012 (8)	0.0095 (9)	−0.0120 (9)
C16	0.0339 (10)	0.0403 (12)	0.0280 (10)	−0.0011 (9)	0.0109 (8)	−0.0078 (9)
C17	0.0267 (9)	0.0329 (10)	0.0235 (9)	0.0014 (8)	0.0053 (7)	−0.0009 (8)

### Geometric parameters (Å, °)

**Table:**

V1—O1	1.589 (1)	C3—H3A	0.9500
V1—O2	1.979 (1)	C4—C5	1.384 (3)
V1—O3	1.992 (1)	C4—H4A	0.9500
V1—O5	2.188 (1)	C5—C6	1.387 (3)
V1—N2	2.071 (1)	C5—H5	0.9500
V1—N4	1.884 (2)	C6—H6	0.9500
O2—C7	1.297 (2)	C8—C10	1.476 (3)
O3—C9	1.304 (2)	C8—C9	1.509 (2)
O4—C9	1.223 (2)	C10—H10A	0.9800
O5—C11	1.252 (2)	C10—H10B	0.9800
N1—C7	1.319 (2)	C10—H10C	0.9800
N1—N2	1.382 (2)	C11—C12	1.473 (2)
N2—C8	1.283 (2)	C12—C13	1.393 (3)
N3—C11	1.341 (2)	C12—C17	1.397 (2)
N3—N4	1.359 (2)	C13—C14	1.382 (3)
N3—H3	0.874 (9)	C13—H13	0.9500
N4—H4	0.879 (9)	C14—C15	1.389 (3)
C1—C2	1.389 (3)	C14—H14	0.9500
C1—C6	1.393 (3)	C15—C16	1.383 (3)
C1—C7	1.481 (2)	C15—H15	0.9500
C2—C3	1.382 (3)	C16—C17	1.385 (3)
C2—H2	0.9500	C16—H16	0.9500
C3—C4	1.389 (3)	C17—H17	0.9500
			
O1—V1—N4	95.10 (7)	C4—C5—H5	120.0
O1—V1—O2	97.53 (7)	C6—C5—H5	120.0
N4—V1—O2	103.39 (6)	C5—C6—C1	120.22 (19)
O1—V1—O3	98.34 (7)	C5—C6—H6	119.9
N4—V1—O3	100.63 (6)	C1—C6—H6	119.9
O2—V1—O3	149.76 (5)	O2—C7—N1	123.88 (16)
O1—V1—N2	110.29 (6)	O2—C7—C1	117.71 (16)
N4—V1—N2	154.60 (6)	N1—C7—C1	118.41 (15)
O2—V1—N2	74.50 (5)	N2—C8—C10	126.85 (16)
O3—V1—N2	75.90 (5)	N2—C8—C9	110.97 (15)
O1—V1—O5	168.87 (6)	C10—C8—C9	122.07 (16)
N4—V1—O5	73.77 (5)	O4—C9—O3	124.44 (16)
O2—V1—O5	85.25 (5)	O4—C9—C8	121.82 (16)
O3—V1—O5	84.11 (5)	O3—C9—C8	113.73 (15)
N2—V1—O5	80.84 (5)	C8—C10—H10A	109.5
C7—O2—V1	116.27 (11)	C8—C10—H10B	109.5
C9—O3—V1	119.54 (11)	H10A—C10—H10B	109.5
C11—O5—V1	113.82 (11)	C8—C10—H10C	109.5
C7—N1—N2	106.71 (14)	H10A—C10—H10C	109.5
C8—N2—N1	121.87 (15)	H10B—C10—H10C	109.5
C8—N2—V1	119.25 (12)	O5—C11—N3	116.36 (15)
N1—N2—V1	118.51 (11)	O5—C11—C12	122.63 (15)
C11—N3—N4	114.16 (14)	N3—C11—C12	121.00 (15)
C11—N3—H3	127.7 (16)	C13—C12—C17	120.13 (16)
N4—N3—H3	118.0 (16)	C13—C12—C11	117.19 (15)
N3—N4—V1	121.87 (11)	C17—C12—C11	122.68 (16)
N3—N4—H4	108.9 (13)	C14—C13—C12	119.97 (17)
V1—N4—H4	129.3 (13)	C14—C13—H13	120.0
C2—C1—C6	119.31 (17)	C12—C13—H13	120.0
C2—C1—C7	120.58 (16)	C13—C14—C15	119.90 (19)
C6—C1—C7	120.11 (17)	C13—C14—H14	120.0
C3—C2—C1	120.43 (18)	C15—C14—H14	120.0
C3—C2—H2	119.8	C16—C15—C14	120.17 (18)
C1—C2—H2	119.8	C16—C15—H15	119.9
C2—C3—C4	120.11 (19)	C14—C15—H15	119.9
C2—C3—H3A	119.9	C15—C16—C17	120.52 (18)
C4—C3—H3A	119.9	C15—C16—H16	119.7
C5—C4—C3	119.82 (19)	C17—C16—H16	119.7
C5—C4—H4A	120.1	C16—C17—C12	119.28 (18)
C3—C4—H4A	120.1	C16—C17—H17	120.4
C4—C5—C6	120.10 (19)	C12—C17—H17	120.4

### Hydrogen-bond geometry (Å, °)

**Table:**

*D*—H···*A*	*D*—H	H···*A*	*D*···*A*	*D*—H···*A*
N3—H3···O4^i^	0.87 (1)	1.97 (1)	2.823 (2)	164 (2)
N4—H4···O3^i^	0.88 (1)	2.05 (1)	2.861 (2)	154 (2)

Symmetry codes: (i) −*x*+3/2, *y*−1/2, −*z*+3/2.
